# Strategy for surgical treatment of congenital subglottic stenosis in children

**DOI:** 10.1007/s00383-012-3134-2

**Published:** 2012-08-29

**Authors:** Mitsumasa Okamoto, Eiji Nishijima, Akiko Yokoi, Makoto Nakao, Yuko Bitoh, Hiroshi Arai

**Affiliations:** Department of Surgery, Kobe Children’s Hospital, Kobe, 654-0081 Japan

**Keywords:** Congenital subglottic stenosis, Laryngotracheoplasty, Costal cartilage graft, KTP laser, Tracheal opening retainer

## Abstract

**Background/purpose:**

Congenital subglottic stenosis is a rare anomaly caused by thickened cricoid cartilage. We report our surgical techniques, comprising anterior cricoid split (ACS), laryngotracheoplasty (LTP), KTP laser ablation, and application of a tracheal opening retainer (TOR) into the tracheostomy site.

**Methods:**

Nine patients have been treated since 1988. Four patients (median age 85 days; range 5 days to 6 months) underwent ACS. Another four patients (median age, 17 months; range, 5–57 months) underwent LTP using costal cartilage grafts, although two had undergone tracheostomy before LTP. One patient underwent LTP, ablation of the projecting part of the cricoid cartilage with KTP laser (LTP + Laser) and, preservation of the tracheal opening by placement of the TOR.

**Results:**

All ACS and LTP patients were successfully extubated at a median of 32 days (range 23–91 days) and 23 days (range 6–31 days) postoperatively, respectively. The LTP + Laser patient was extubated 35 days after surgery and the TOR was removed asymptomatically 20 days after extubation of the stent tube.

**Conclusions:**

Anterior cricoid split is useful for patients ≤6 months old and LTP is useful for patients >6 months old and/or with tracheostomy. KTP laser ablation is effective to remove thickened parts of cricoid cartilage protecting the vocal cords. The tracheal opening preserved by the TOR works as an additional channel to safeguard respiration during the extubation process.

## Introduction

Congenital subglottic stenosis (CSS) is a rare anomaly caused by thickened cricoid cartilage. Several surgical techniques have been introduced [1], but outcomes of these procedures remain unclear, because CSS is rare and few reports have described surgical results. The aim of this study was to clarify outcomes of our surgical management and to outline our surgical strategy for CSS, which includes applications of both KTP laser ablation for the thickened part of the cricoid cartilage and placement of a tracheal opening retainer (TOR) to secure an alternative airway at extubation.

## Materials and methods

This study was undertaken with the approval of the institutional review board (registration no. R23-4) and informed consent was obtained from the guardian of each patient prior to surgery. Since 1988, a total of nine patients with CSS have been treated at our institution. Medical charts were reviewed retrospectively and patients’ characteristics are summarized in Table 1. In all cases, stridor was found in the neonate without upper airway infection before the first intratracheal intubation for respiratory care. Difficulty of intratracheal intubation was pointed out at the first intubation in all cases. The diagnosis was confirmed by rigid bronchoscopy in the operating room under general anesthesia. Annular or iris-like stenosis was observed at the level of the cricoid cartilage. Severity of subglottic airway stenosis was graded according to the Myer–Cotton classification system: grade I, to 50 % stenosis; grade II, from 51 to 70 %; grade III, from 71 to 99 %, or grade IV, no identifiable lumen present [2].

**Table 1:** Age, severity of stenosis, previous tracheostomy, type of surgery,timing of extubation, and follow-up in nine CSS cases

Case	Age at surgery	Severity of stenosis^a^	Previous tracheostomy	Surgical technique	Timing of extubation (days)	Symptoms after extubation (follow-up)
1	6 months	Grade III	No	ACS	35	Asymptomatic (22 years and 6 months)
2	3 months	Grade I	No	ACS	91	Asymptomatic (21 years and 6 months)
3	5 days	Grade I	No	ACS	23	Asymptomatic (20 years and 7 months)
4	1 month	Grade I	No	ACS	28	Dysphonia by paralysis of the bilateral vocal cords at birth (2 years and 2 months)
5	5 months	Grade II	Yes	LTP	31	Asymptomatic (23 years and 2 months)
6	1 year and 7 months	Grade II	Yes	LTP	28	Asymptomatic (11 years and 5 months)
7	4 years and 9 months	Grade II	No	LTP	6	Recurrent bronchitis caused by Kartagener syndrome (6 years and 10 months)
8	1 year and 3 months	Grade II	No	LTP	27	Asymptomatic (4 years and 10 months)
9	5 years and 8 months	Grade II	Yes	LTP + Laser	35^b^, 55^c^	Asymptomatic (2 years and 10 months)

*ACS* anterior cricoid split, *LTP* laryngotracheoplasty

^a^Graded according to the Myer–Cotton classification

^b^Extubation of nasotracheal stent tube

^c^Removal of TOR

Anterior cricoid split (ACS) [3] was applied to four patients (cases 1–4; median age 85 days; range 5 days to 6 months). After exposing the anterior surface of the larynx with a small collar incision, a midline incision was performed anteriorly in the larynx and upper part of the trachea extending from the middle of the thyroid cartilage down to the first or second tracheal cartilage ring. A nasotracheal stent tube of one-size larger caliber than the usual one was left in place without any resection of the thickened parts of the cricoid cartilage.

**Fig. 1:**
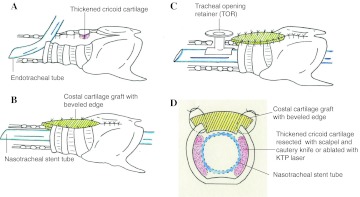
Laryngotracheoplasty (LTP) using costal cartilage graft. **a** An endotracheal tube was intubated through the tracheal split in the operative field. Thickened cricoid cartilage in the lumen was resected with scalpel and cautery knife or was ablated with KTP laser. **b** Autologous costal cartilage graft with beveled edge was sutured into the position with 5-0 monofilament absorbable sutures after a nasotracheal stent tube was inserted and fixed. **c** The tracheal opening was preserved by placing of a tracheal opening retainer (TOR). **d** A cross section of the subglottic area on LTP

Laryngotracheoplasty (LTP) [4] using costal cartilage graft was applied to four patients (cases 5–8; median age 17 months; range 5–57 months), although two had undergone creation of a tracheostomy before the procedure. An anterior midline incision of the larynx was performed in the same manner as ACS. An endotracheal tube was temporarily intubated through the split tracheal wall in the operative field during the procedure for ventilation. The thickened parts of cricoid cartilage in the lumen were resected with a scalpel and/or cautery knife as much as possible. Costal cartilage with intact perichondrium was then resected from the sixth or seventh costal arch. This autologous cartilage graft was carved into a spindle shape with beveled edges to avoid dislodgement into the laryngeal lumen. Immediately after the removal of the endotracheal tube, a nasotracheal stent tube of adequate caliber was carefully inserted and fixed to maintain artificial ventilation. The cartilage graft was interruptedly sutured to the split anterior edges of the cricoid with 5-0 monofilament absorbable sutures. In cases with tracheostomy, the midline incision was extended down to the tracheostomy site and the tracheal wall was closed using the graft. Postoperative ventilation was maintained only through the nasotracheal stent tube (Fig. 1a, b), with the patient breathing spontaneously via stent tubes after recovery from artificial ventilation.

In one patient (case 9), LTP was performed at 5 years of age. This LTP consisted of ablating thickened cricoid cartilage with a KTP laser by direct manipulation in the operative field exposed by a laryngotracheal midline incision (LTP + Laser; Fig. 1a, c) and preserving the tracheal opening using the TOR (KOKEN, Tokyo, Japan) [5], a soft silicone cuffed button-like cannula.

Rigid endoscopy or bronchofiberscopy was regularly performed in all nine cases before extubation. Systemic administration of steroids was started after extubation and maintained for several weeks in an every-other-day fashion, to prevent edema and granulation in the larynx.

## Results

All nine patients were successfully extubated. The four ACS patients (cases 1–4) were extubated at a median of 31.5 days (range, 23–91 days) postoperatively. A rather long time was required for case 2 to be extubated because of recurrent hypoxemia caused by bronchomalacia of the right main bronchus, which had been identified before ACS. The four LTP patients (cases 5–8) were successfully extubated at a median of 22.5 days (range 6–31 days) postoperatively.

**Fig. 2:**
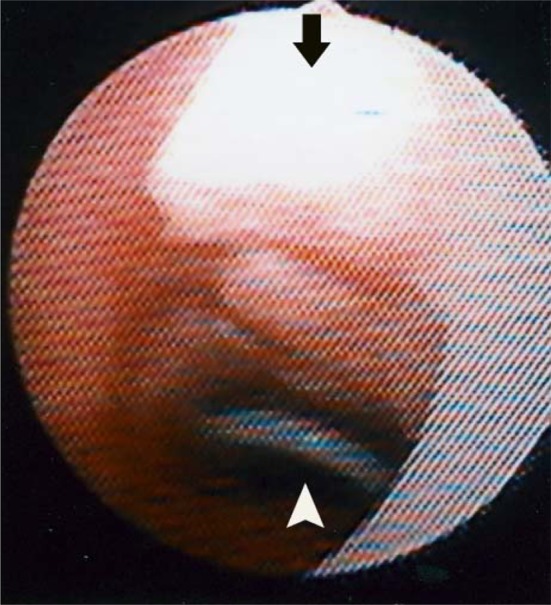
Widening of the subglottic lumen, inner surface of sutured costal cartilage graft (*arrow*) and inner flange of TOR (*arrowhead*) were observed at bronchoscopy when the nasotracheal stent tube was removed partly in case 9

In the LTP + Laser patient (Case 9), extubation of the nasotracheal stent tube was performed 35 days postoperatively. Before removal of the stent tube, widening of the subglottic lumen and full epithelialization of the inner surface of the grafted cartilage were confirmed by bronchoscopy (Fig. 2). The patient was able to breathe through both the larynx and TOR immediately after extubation without any trouble. The TOR was removed another 20 days after removal of the stent tube. No symptoms have been observed since TOR removal.

The nine cases were followed for a median of 11 years and 5 months (range 2–23 years) after extubation. Case 4 was successfully extubated 28 days postoperatively, but dysphonia remained present due to congenital paralysis of bilateral vocal cords. Case 7 was successfully extubated 6 days postoperatively, because preoperative stenosis was limited. However, this patient has been suffering from recurrent bronchitis caused by Kartagener syndrome, which was identified later (Table 1).

## Discussion

Historically, several methods of laryngotracheal reconstruction have been performed [1]. Outcomes for methods such as ACS and LTP have been reported [6, 7], but have mostly been performed on patients with acquired subglottic stenosis. As CSS is a rare disease, no definitive reviews of surgical outcomes for patients with CSS have yet been reported.

ACS [3] has been used for many years and involves placement of an endotracheal stent tube to support the split part of the anterior cricoid wall during healing. This operation was originally applied to acquired subglottic stenosis in the premature infant and was successfully managed without leakage. In the present series, we performed this technique on four patients who were ≤6 months old, with successful extubation in all cases. Due to structural plasticity and the small size of the larynx in these infants, an endotracheal stent tube one-size larger than usual should be placed to enlarge the subglottic lumen without causing leakage.

LTP [4] has been the most available treatment of choice in infants and children with acquired subglottic stenosis. Several types of materials have been used for grafts [8–12], with costal cartilage as the most common tissue used successfully in LTP. Four patients who were ≥6 months old and/or had undergone previous tracheostomy underwent ordinary LTP using costal cartilage grafting and all four were successfully extubated. We applied LTP to the two patients who had undergone previous tracheostomy, because granulation and cicatrization of the trachea proximal to the tracheostomy should be repaired simultaneously at surgery.

For case 9, we tried ablating the thickened parts of the cricoid cartilage with KTP laser and preserving the tracheal opening by inserting the TOR when LTP with costal cartilage grafting was performed. The nasotracheal stent tube was extubated later than usual, because the outcome of ablating thickened cricoid cartilage with KTP laser was not clearly predictable. Endoscopic laser ablation has been reported as ineffective on the thickened cricoid cartilage [13]. In our experience, we found that ablation with KTP laser under direct vision through a midline laryngofissure was safe and effective to precisely vaporize the thickened cricoid cartilage while preserving the vocal cords.

Retaining the tracheal opening by TOR at LTP was effective for safely providing an alternative channel for respiration during extubation of the nasotracheal stent tube. We can use the TOR without disturbing the placement of the nasotracheal stent tube at LTP because of the flexibility and shortness of the TOR flanges. The TOR is easily removed at any time immediately before insertion of a tracheostomy tube [5].

We have successfully managed a series of patients with CSS, which is caused by thickened cricoid cartilage with no or very low levels of inflammation. However, acquired subglottic stenosis is more difficult to cure than CSS, as such lesions are caused by inflammation such as edema, granulation, and scar tissue due to irritable intubation into the larynx. This inflammation often extends to the supraglottic, glottic, and subglottic areas, and successful treatment of the larynx without recurrence due to granulation tissue in the same fashion as CSS remains difficult. We consider that retention of the tracheal opening by TOR at LTP is also important when treating children with acquired subglottic stenosis, and long-term T-tube stenting is recommended as the initial treatment if the stenosis is severe [14].
